# Supplementary material for: Multi‐dataset identification of innovative feature genes and molecular mechanisms in keratoconus
Source: J Cell Mol Med. 2024 Sep 19;28(18):e70079. doi: 10.1111/jcmm.70079 (PMC11412914; doi:10.1111/jcmm.70079)
Supplement: Supplementary file 2 — Material S2. [file JCMM-28-e70079-s002.zip › 7.GSEA analysis/6.SNORA74B/KEGG_DNA_REPLICATION.html]

Details for gene set KEGG\_DNA\_REPLICATION[GSEA]

|  || Dataset | SNORA74B.group.cls#h\_versus\_l |
| Phenotype | group.cls#h\_versus\_l |
| Upregulated in class | l |
| GeneSet | KEGG\_DNA\_REPLICATION |
| Enrichment Score (ES) | -0.41315177 |
| Normalized Enrichment Score (NES) | -1.3836533 |
| Nominal p-value | 0.115530305 |
| FDR q-value | 0.3388785 |
| FWER p-Value | 0.912 |
Table: GSEA Results Summary

  

Fig 1: Enrichment plot: KEGG\_DNA\_REPLICATION      
 Profile of the Running ES Score & Positions of GeneSet Members on the Rank Ordered List

  

| SYMBOL | TITLE | RANK IN GENE LIST | RANK METRIC SCORE | RUNNING ES | CORE ENRICHMENT || 1 | RPA4 | na | 613 | 0.566 | 0.1053 | No |
| 2 | DNA2 | na | 7044 | 0.161 | -0.0450 | No |
| 3 | POLE | na | 8968 | 0.114 | -0.0756 | No |
| 4 | RNASEH2B | na | 12695 | 0.051 | -0.1720 | No |
| 5 | SSBP1 | na | 13992 | 0.036 | -0.2015 | No |
| 6 | POLD4 | na | 14153 | 0.034 | -0.1987 | No |
| 7 | POLD1 | na | 14676 | 0.028 | -0.2077 | No |
| 8 | RPA1 | na | 18927 | -0.016 | -0.3267 | No |
| 9 | PCNA | na | 19297 | -0.021 | -0.3328 | No |
| 10 | POLE4 | na | 19561 | -0.024 | -0.3352 | No |
| 11 | RPA2 | na | 22266 | -0.055 | -0.4012 | Yes |
| 12 | POLE3 | na | 22272 | -0.055 | -0.3894 | Yes |
| 13 | POLD2 | na | 22465 | -0.058 | -0.3824 | Yes |
| 14 | RNASEH1 | na | 23304 | -0.070 | -0.3913 | Yes |
| 15 | POLA1 | na | 23506 | -0.073 | -0.3813 | Yes |
| 16 | MCM3 | na | 23664 | -0.076 | -0.3694 | Yes |
| 17 | POLD3 | na | 23900 | -0.079 | -0.3589 | Yes |
| 18 | PRIM2 | na | 24056 | -0.082 | -0.3456 | Yes |
| 19 | LIG1 | na | 24194 | -0.084 | -0.3314 | Yes |
| 20 | RNASEH2A | na | 24301 | -0.086 | -0.3158 | Yes |
| 21 | RPA3 | na | 25111 | -0.100 | -0.3173 | Yes |
| 22 | MCM7 | na | 25197 | -0.102 | -0.2976 | Yes |
| 23 | RFC5 | na | 25360 | -0.105 | -0.2795 | Yes |
| 24 | RNASEH2C | na | 26190 | -0.121 | -0.2771 | Yes |
| 25 | RFC3 | na | 26890 | -0.137 | -0.2674 | Yes |
| 26 | RFC2 | na | 26940 | -0.138 | -0.2388 | Yes |
| 27 | RFC4 | na | 27030 | -0.140 | -0.2109 | Yes |
| 28 | MCM4 | na | 27082 | -0.141 | -0.1816 | Yes |
| 29 | RFC1 | na | 27659 | -0.156 | -0.1644 | Yes |
| 30 | POLA2 | na | 29251 | -0.202 | -0.1663 | Yes |
| 31 | MCM5 | na | 29445 | -0.208 | -0.1266 | Yes |
| 32 | PRIM1 | na | 29730 | -0.216 | -0.0877 | Yes |
| 33 | MCM6 | na | 30422 | -0.242 | -0.0550 | Yes |
| 34 | FEN1 | na | 30485 | -0.245 | -0.0036 | Yes |
| 35 | MCM2 | na | 31109 | -0.274 | 0.0380 | Yes |
| 36 | POLE2 | na | 31670 | -0.305 | 0.0881 | Yes |
Table: GSEA details [plain text format]

  

Fig 2: KEGG\_DNA\_REPLICATION      
 Blue-Pink O' Gram in the Space of the Analyzed GeneSet

  

Fig 3: KEGG\_DNA\_REPLICATION: Random ES distribution      
 Gene set null distribution of ES for **KEGG\_DNA\_REPLICATION**
